# Transcriptome data reveal gene clusters and key genes in pepper response to heat shock

**DOI:** 10.3389/fpls.2022.946475

**Published:** 2022-09-21

**Authors:** Bingqian Tang, Xiumin Li, Xinhao Zhang, Qinbiao Yin, LingLing Xie, Xuexiao Zou, Feng Liu, Xiongze Dai

**Affiliations:** ^1^College of Horticulture, Hunan Agricultural University, Changsha, China; ^2^Longping Branch, Graduate School of Hunan University, Changsha, China; ^3^ERC for Germplasm Innovation and New Variety, Breeding of Horticultural Crops, Changsha, China; ^4^Key Laboratory for Vegetable Biology of Hunan Province, Changsha, China

**Keywords:** pepper, heat stress, gene expression, heat shock factors (HSFs), clustering analysis

## Abstract

Climate change and global warming pose a great threat to plant growth and development as well as crop productivity. To better study the genome-wide gene expression under heat, we performed a time-course (0.5 to 24 h) transcriptome analysis in the leaf and root of 40-day-old pepper plants under 40°C as well as in control plants. Clustering analysis (K-means) showed that the expression of 29,249 genes can be grouped into 12 clusters with distinct expression dynamics under stress. Gene ontology (GO) enrichment analysis and transcription factor (TF) identification were performed on the clusters with certain expression patterns. Comparative analysis between the heat-treated and control plants also identified differentially expressed genes (DEGs), which showed the largest degree of change at 24 h. Interestingly, more DEGs were identified in the root than in the leaf. Moreover, we analyzed the gene expression of 25 heat shock factor genes (HSFs) in pepper after heat stress, identified five of these HSFs that responded to heat stress, and characterized the role of these genes in heat-tolerant (17CL30) and heat-susceptible (05S180) pepper lines. The findings of this study improve our understanding of the genome-wide heat stress response in pepper.

## Introduction

Global climate change makes extreme weather more frequent, presenting ever-increasing environmental stress on plants. Such stress dysregulates the normal growth and development in plants and is a major threatening factor for food safety (Fedoroff et al., 2010; Zhu, 2016; Jagadish et al., 2021). Among them, high-temperature stress is a significant challenge facing plants because an increase of 10–15°C in temperature would threaten the survival of plants (Lipiec et al., 2013). Indeed, heat stress has a significant negative impact on the growth and productivity of plants by inhibiting multiple biological processes such as photosynthesis, absorption of minerals, pollination, and seed germination of seeds (Nayyar et al., 2013; Bahuguna et al., 2016; Ali et al., 2019). To have a better understanding of how plant adapts to heat stress (HS), and to facilitate the process of breeding for heat tolerance, it is important to investigate the molecular mechanisms underlying the phenotypic changes when the plant was under HS treatment. The mechanism of plant sensing and subsequently responding to HS, and the complex transcriptional regulatory network involved in the sense and response processes have been widely studied (Ohama et al., 2017; Zhang et al., 2022). Recently, studies in rice have revealed for the first time that complex quantitative trait loci controlled HS resistance. Following *TT1* (Li et al., 2015) and *TT2* (Kan et al., 2022), a new rice HS resistance locus *TT3* was identified and the two constituent genes, *TT3.1* and *TT3.2*, were isolated and cloned. The ubiquitin ligase activity of TT3.1 induces the degradation of TT3.2, a chloroplast precursor protein that triggers chloroplast damage in the context of heat stress. Thus, these two genes interact antagonistically to enhance HS tolerance in rice (Zhang et al., 2022).

Plants have evolved a series of adaptive mechanisms to cope with long-term heat stress (Baurle, 2016). Heat stress activates heat shock factors (HSFs), and HSFs play critical roles in regulating the expression of heat-responsive genes (Tong et al., 2022), which further induce the expression of heat shock proteins (HSPs) to mediate the heat shock response (HSR) (Hu et al., 2009). Previous studies had shown that HSFs are the core components for effective protection against heat (Xue et al., 2014), but through gene transcription while HSPs are responsible for protein folding, assembly, translocation, and degradation (Molinier et al., 2006). As the terminal components of signal transduction, HSFs mediate the gene expression networks to various abiotic stresses (Nover et al., 2001; Scharf et al., 2012; Guo et al., 2016). Some studies in wheat have shown that Hsf may have the function of regulating wheat to cope with multiple stresses (Duan et al., 2019). Recent studies have shown that the wheat *TaHsfA2-13* plays an important role in responding to a variety of abiotic stresses and phytohormones (Meng et al., 2022). Related studies on the identification of *HsfA2* in tomato and pepper (Guo et al., 2014a,^2015^; Fragkostefanakis et al., 2016). In addition, genes such as *CaHSP70-1* (Guo et al., 2014b), *CaHSP25.9* (Feng et al., 2019), and *CaHSP22.0* (Sun et al., 2019) were identified in pepper, which have positive regulatory effects on improving the high-temperature resistance of pepper.

*Capsicum annuum* L. is native to the tropical regions of Central and South America with an optimum growth temperature of 18 to 30°C (Olatunji and Afolayan, 2018). However, it is not heat-resistant as exposure to high temperature (above 32°C), leading to multiple heat stress symptoms such as abortion of the pollen, decrease in the number of flower and fruit, and reduction in pepper yield. Therefore, global warming and high temperature in summer could be detrimental to pepper yield and quality (Lee et al., 2018). It is critical to understand the molecular mechanisms underlying heat responses for selective breeding of heat-tolerant plants. To this end, a previous study assessed heat tolerance in pepper germplasms and identified a heat-tolerant variety (17CL30) and a heat-susceptible variety (05S180) (Wang et al., 2019). Furthermore, the authors profiled the metabolome and transcriptome of 17CL30 and 05S180 under normal conditions and high-temperature stress and identified HSR as an important heat-resistant mechanism. This study highlighted the importance of omics in understanding the molecular mechanisms of heat responses. In addition, it also provided a rich data source to mine key genes regulating homeostasis under heat. However, the previous study is limited in that single time point of leaf samples in heat-resistant and heat-susceptible varieties were used. Thus, it is necessary to profile the time-course gene expression of multiple tissues in normal pepper plants.

In this study, 40-day-old hydroponic pepper plants were subjected to 40°C heat. A time-course comparative transcriptomics analysis was performed using both leaf and root samples (Liu et al., 2017). Thus, a gene regulatory network under heat stress in pepper was constructed. Both time-dependent and tissue-dependent differentially expressed genes (DEGs) under heat stress were identified. Gene ontology (GO) analysis and Kyoto Encyclopedia of Genes and Genomes (KEGG) pathway enrichment analysis of these DEGs revealed important mechanisms underlying the heat response in pepper. Using K-means clustering, pepper genes expressed in response to HS on the genome-wide level were grouped into clusters based on similar biological functions or similar regulation. This facilitated the identification of co-functional genes, including transcription factors, which are downstream elements of signal transduction and play an indispensable role in the regulation of plant genes in response to abiotic stresses. We analyzed the transcription factors included in clusters 2 and 6, which were more clearly responsive to HS. In addition, we identified *CaTT3.1*, the ortholog of the rice HS-responsive gene *TT3.1* (Zhang et al., 2022), from cluster 2 and found that the expression of this gene was significantly upregulated after pepper was exposed to HS for 6 h. In addition, the expression profiles of HSF genes and their responses to heat in pepper were further determined.

## Materials and methods

### Plant materials and data sources

Total RNA from different pepper tissues was prepared as described previously (Liu et al., 2017). An elite breeding line of *C. annuum*, the inbred line “6421” (hereafter referred to as line 6421), was selected from a pepper landrace (long and red in phenotype) widely grown on the west side of Xiang Jiang River, Hunan Province, China.

The seedlings were grown at 25/18°C day/night temperature, and with a 16/8 light/dark cycle, relative humidity of 60–70%, and light intensity of 6000 Lux. For heat treatment, the seedlings were transferred into a growth chamber of 42°C, and the illumination, photoperiod, and relative humidity were kept the same as plants without the heat treatment. Leaf and root tissues were collected from both treated and control plants at 0.5, 1, 3, 6, 12, and 24 h post-treatment (HPT). Considering the effect of the circadian clock on plant gene expression, samples were collected at 8:30, 9:00, 11:00, 14:00, and 20:00 on the first day and 8:00 on the next day. A total of 24 samples (six time points for two types of tissue: leaf and root) were collected. Each sample had three biological repeats, and each repeat consisted of leaf or root tissues from five randomly selected seedlings. For each sample, averaged data from the three biological repeats were shown.

### Measurements of the conductivity and the contents of chlorophylls, reactive oxygen species, and proline

The contents of chlorophylls, reactive oxygen species (ROS), and proline and the conductivity were measured in the leaf samples at 0, 6, 12, and 24 HPT. The contents of proline, ROS, and chlorophylls were measured by the methods described in Bates et al. (1973), Zhang et al. (2008), and Kim and Xue (2020), respectively. For the conductivity measurement, air in the intercellular space of pepper leaves was extracted using a vacuum dryer and an aspirator, and then, the leaves were hydrated by soaking in water. The conductivity of the solution was measured with a conductivity meter. All the above-mentioned measurements were conducted with three biological repeats for each sample.

### Differential gene expression analysis

Quality control of the dataset was performed using fastp (Chen et al., 2018) and FastQC v0.11.7 (Andrews, 2017). Next, the data were aligned to the reference genome of pepper (Zunla genome) (Qin et al., 2014). Mapping was performed using HISAT 2.2.1 with default parameters (10 mismatches/read; nine multi-mapping locations/read) (Kim et al., 2015). The abundance of a transcript was measured as the mean normalized count of reads mapping onto the transcript (Love et al., 2014). The genome index was generated using the gene annotation file (gff file).

Differential gene expression analysis was carried out using DESeq2 v1.20.0, an R-based package available from Bioconductor (Love et al., 2014). The abundance of transcripts was used to identify DEGs between two conditions. The ratio of mean normalized counts between two conditions was log-transformed [log fold change, or log2(FC)]. Up- or downregulated DEGs were defined using the following criteria: |log2(FC)| ≥ 2 and adjusted *p*-value < 0.01. A statistical test was performed using the negative binomial Wald test followed by a Benjamini-Hochberg correction to obtain the adjusted *p*-value or false discovery rate (FDR). Venn diagrams were created as described (Lin et al., 2016). Heatmaps were generated using Python (seaborn heatmap). Summary statistics on the sequencing data are provided in Supplementary Table 1. DEGs identified in the leaf and root at each time point are available in Supplementary Table 6.

### Clustering and gene enrichment analyses

Clustering analysis was performed on 12 control and 12 heat-treated samples using the k-means method in Python (Supplementary Table 2; Gasch and Eisen, 2002). For each gene, the normalized expression was calculated by dividing the expression levels from all samples with its maximum observed transcripts per million (TPM). Hierarchical clustering (HCL) and principal component analysis (PCA) were performed using the kernel PCA method in Python with default settings. Identified clusters responsive to heat stress were further subjected to gene enrichment with GOATOOLS (Klopfenstein et al., 2018) and a Python package for GO analysis. Statistically significant GO terms were defined using FDR < 0.05. The KEGG pathway analysis was performed by KofamKOALA (Aramaki et al., 2020), and enrichment analysis was carried out using R package clusterProfiler (Yu et al., 2012).

### Identification of heat shock factor genes

The HSF domain was used for searching homologous genes in the tomato and pepper genomes. In total, 26 and 25 HSF genes were identified in tomato and pepper, respectively. For the identification of transcription factors (TFs), the iTAK database and associated rules were used^1^ (Zheng et al., 2016). Final validation of potential HSFs was performed by comparing the list to that in the PlantTFcat (Dai et al., 2013) and PlnTFDB (Perez-Rodriguez et al., 2010).

### Phylogenetic and homologous alignment analysis

The proteome databases for Lycopersicon esculentum (ITAG3.2) and C. annuum L. (Zunla) were downloaded from NCBI.^2^ Protein sequences from the primary transcripts were used for the construction of orthogroups between the two species using OrthoMCL.^3^ Multiple sequence alignment of Capsicum and Arabidopsis genes was conducted using MUSCLE (Multiple Protein Sequence Alignment) under Linux. Phylogenetic trees were constructed using Tree Best software with the contiguous algorithm (Edgar, 2004) and illustrated using the online tool iTOL (Interactive Tree Of Life)^4^ (Letunic and Bork, 2016).

The gene *CaTT3.1*, orthologous to the rice gene *TT3.1*, was identified by homologous alignment of protein sequences of the two genes, followed by comparing their motifs predicted by MEME (Buske et al., 2010).^5^

### cDNA generation and quantitative/real-time PCR analysis

The total RNA was isolated from the leaf of capsicum “6421” using RNAiso Plus reagent (TaKaRa) at 0, 6, 12, and 24 h after 42°C heat stress and treated with RNase-free DNase I (Promega) and HiScript II 1st Strand cDNA Synthesis kit (Vazyme). qPCR was performed using LightCycler 96 (Roche) with the SYBR Green Premix Ex Taq™ II quantitative PCR system (TaKaRa, Dalian, China). The primers of *CaUBI3* and ten genes are listed in Supplementary Table 12. The ΔCT method was used to calculate the relative expression levels of these genes (Tang et al., 2021) based on the following equation: ΔCT = [Gene expression–mean (*CaUBI3* expression)]/3.

## Results

### Transcriptomics analysis of pepper plants under heat stress

**FIGURE 1:**
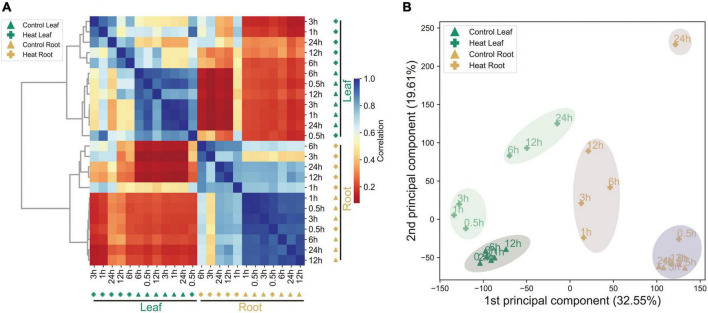
Summary of the transcriptomics data. **(A)** Hierarchical clustering analysis of 35,336 genes from 24 samples. Color scale represents Pearson’s correlation coefficient (0–1). **(B)** PCA of the leaf and root transcriptomes from the control and heat-treated samples.

To study heat stress responses in pepper at the transcript level, a time-course transcriptome analysis of both leaf and root tissues in pepper plants was performed and the normalized gene expression profiles were shown (Figure 1A). Sample data including all biological replicates are recorded in Supplementary Table 1. As expected, the overall expression showed a tissue-dependent pattern, as revealed by both Pearson’s correlation analysis (Figure 1A) and PCA (principal components analysis) (Figure 1B). Interestingly, samples with shorter exposure to heat (0.5 to 3 h) were clustered together with the control, while samples under longer exposure (6 to 24 h) clustered together, indicating significant changes in gene expression after 6 h of treatment. By contrast, transcriptomics reprogramming was observed as early as 1 h after treatment, indicating a faster response in roots compared to that in leaves following heat treatment. Moreover, a drastic change in gene expression was observed even at 24 HPT. Thus, these data suggest heat significantly induces transcriptomics reprogramming in both leaves and roots with different levels of dynamics.

**FIGURE 2:**
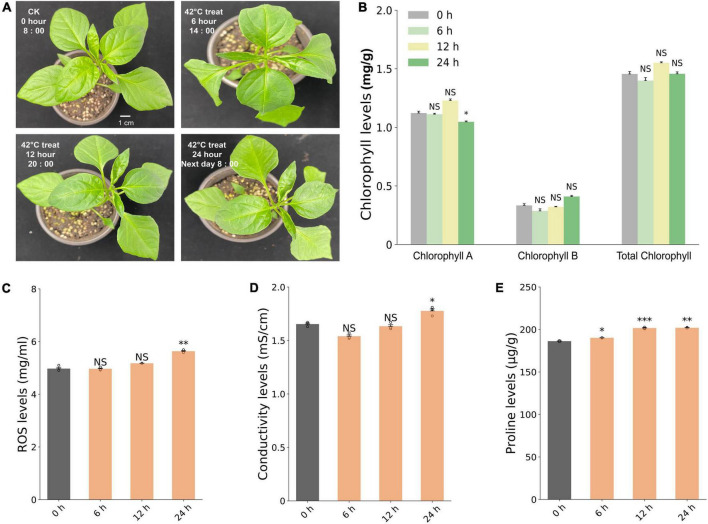
Phenotypic and physiological responses of pepper line 6421 under 42°C heat stress. **(A)** Seedlings of control (0 h) and heat (6, 12, and 24 h at 42°C) treatments. Changes in the content of chlorophyll including chlorophyll A, chlorophyll B, and total chlorophylls **(B)**, the content of ROS **(C)**, conductivity **(D)**, and the content of proline **(E)**. Student’s *t*-test was conducted to compare each heat treatment time point with the 0-h control treatment. Stars indicate significance with one, two, and three stars representing significance at *P < 0.05, **P < 0.01, and ***P < 0.001, respectively. NS: no significant difference. Error bar represents the standard deviation of each mean (*n* = 3).

We conducted phenotypic examination and measurement of physiological parameters, including level of conductivity and contents of chlorophylls, ROS, and proline in the seedlings of the pepper line 6421 after heat treatment at 42°C for 0, 6, 12, and 24 h (Figure 2). No macroscopic wilting was found on pepper leaves at all the investigated time points (Figure 2A). In contrast, the measured physiological parameters were significantly changed after HS, compared to the 0-h control. At 24 HPT, the content of chlorophyll A in leaves decreased significantly, albeit chlorophyll B and total chlorophyll did not change (Figure 2B). Both the ROS content and the conductivity level increased significantly at 24 HPT (*P* < 0.01) (Figure 2C,D). Proline content started to increase significantly at 6 HPT (*P* < 0.05) and the increasing continued at 12 (*P* < 0.001) and 24 HPT (*P* < 0.01) (Figure 2E).

### Tissue-dependent clustering analysis reveals distinct gene expression patterns under heat

**FIGURE 3:**
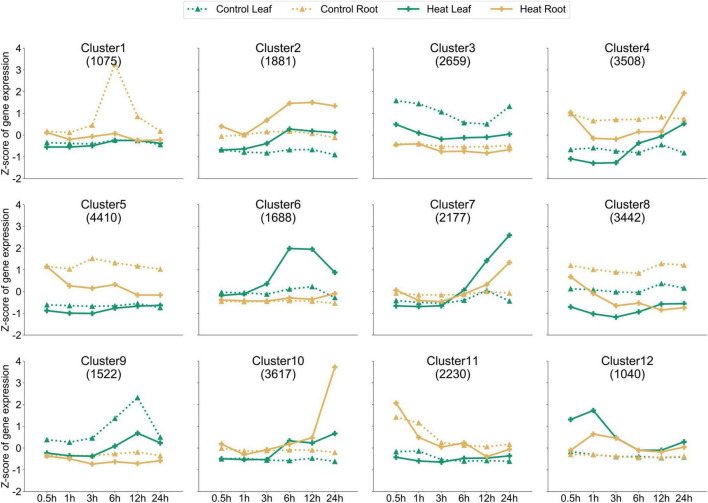
Dynamic gene expression. Twelve clusters (by k-means clustering) with different numbers of genes (shown under the cluster identification) are shown. The *x*-axis depicts six time points, and the *y*-axis depicts the standardized Z-score per gene. Dotted and solids lines (green for the leaf and orange for the root) denote the control and treated samples, respectively.

Based on the expression of 29,249 genes, the k-means clustering algorithm was used to further understand the dynamic patterns of gene expression modulation under heat, which revealed 12 distinctive clusters (Figure 3 and Supplementary Table 2). Among them, the solid lines (HS-treated leaf or root) of clusters 2, 6, 7, 10, and 12 were higher than the corresponding dotted lines, indicating that these clusters contained genes upregulated by HS at the investigated time points. On the contrary, in clusters 1, 3, 5, 8, and 9, the solid line was lower than the dashed line, indicating that these clusters contain genes downregulated by HS at the investigated time points. For example, 1,881 genes in cluster 2 showed much higher expression levels in roots at 6 and 24 HPT compared to those in control. A similar trend was found for cluster 6 genes in the leaf. On the contrary, genes in clusters 3 and 9 (leaf) and cluster 8 (both leaf and root) showed reduced expression following heat treatment. Thus, our dataset provides rich information on the heat-inducible gene expression dynamics, which can be used to understand the molecular mechanisms by which pepper responds to heat.

**FIGURE 4:**
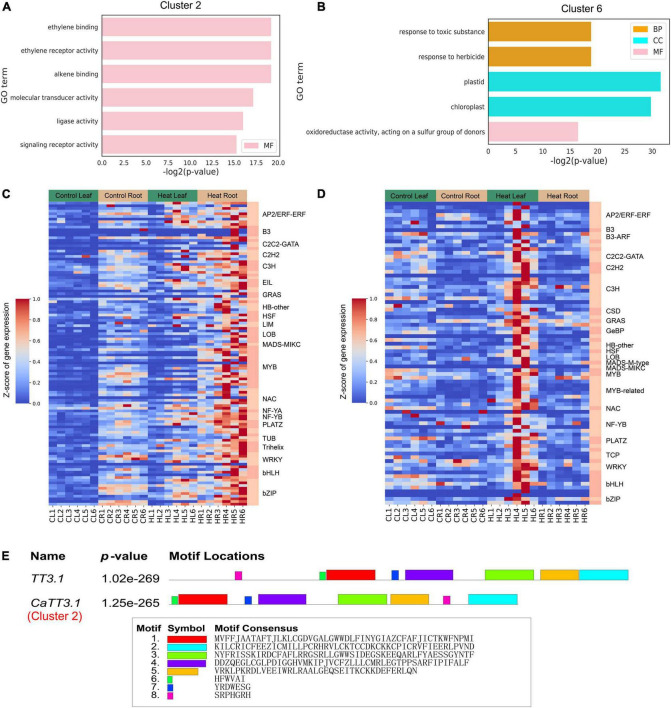
Gene ontology (GO) and heat-responsive transcriptional modulators of Cluster 2 and 6. **(A,B)** GO enrichment results in cluster 2(A) and cluster 6(B). GO terms with FDR < 0.01 are shown. CC, cellular component; MF, molecular function; BP, biological process. The full lists of DEGs and GO terms can be found in Supplementary Tables 3, 4. **(C,D)** Distribution and expression of transcription factors identified in cluster 2(C) and cluster 6(D). **(E)** Alignment of *TT3.1* and *CaTT3.1* protein sequences. The conserved motifs are highlighted and listed.

According to the clustering situation in Figure 3, we further performed gene enrichment analysis on the genes in clusters 2 and 6 and found that the GO terms in cluster 2 (Figure 4A) were mainly enriched in ethylene binding, ethylene receptor activity, alkene binding, molecular transducer activity, ligase activity, and signaling receptor activity; and the GO terms in cluster 6 are mainly in response to toxic substance and herbicide, oxidoreductase activity (Figure 4B). In both clusters 2 and 6, the expression level in roots and leaves 6 h after heat stress was higher than that in untreated ones, among which cluster 2 was increased in both leaves and roots, while cluster 6 was only increased in leaves, so these two genes in each cluster have different expression patterns. Finally, 114 and 84 transcription factors were identified in cluster 2 (1881 genes) and cluster 6 (1688 genes), respectively, and their distributions are shown in Figure 4C (Cluster 2) and Figure 4D (Cluster 6). The most identified transcription factor family was the MYB family, with 16 and 10 MYB family members identified, respectively. A recent study suggested that the rice gene *TT3.1* might be a potential thermosensor (Zhang et al., 2022). In this study, comparing the protein sequences of *TT3.1* and its pepper ortholog *CaTT3.1* identified five large segments of motif conservation (Motif 1-5 in Figure 4E). *CaTT3.1* was clustered in the Cluster 2, in which all genes were significantly upregulated in roots and leaves at 6 HPT and thereafter. These results suggested that *CaTT3.1* is related to the HS response and may be functional in pepper as the counterpart of *TT3.1* in rice. However, study on functional verification of *CaTT3.1* is yet to be conducted.

### Identification of differentially expressed genes under heat stress

**FIGURE 5:**
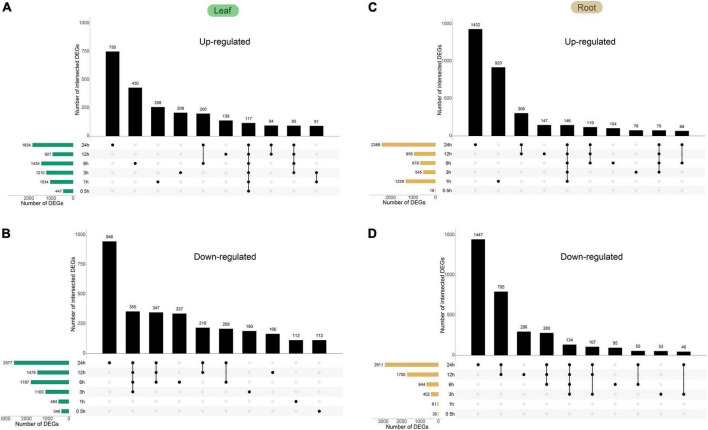
Time-course transcriptomics analysis of Capsicum annuum L. under heat. Plants were subjected to 42°C treatment for the indicated time. The total numbers of significant genes at each time point are shown on the left. **(A)** Upregulated genes in the leaf. **(B)** Downregulated genes in the leaf. **(C)** Upregulated genes in the root. **(D)** Downregulated genes in the root. All the DEGs in roots and leaves are shown in Supplementary Table 5.

Next, we identified DEGs under heat stress (24 h at 42°C) by a comparative analysis, which revealed 7,318 DEGs in the leaf (3,490 and 3,828 for up- and downregulated, respectively) and 7,271 DEGs in the root (3,791 and 3,480 for up- and downregulated, respectively). While a small fraction of genes showed opposite trends in regulation at different time points after treatment (73 in the leaf and 150 in the root), the majority of DEGs showed the same trend across the time course. Collectively, 9,981 DEGs were identified from either tissue type, constituting ∼34.1% of all expressed genes in the dataset. The comparative analysis also revealed several trends in transcriptional reprogramming under heat in pepper plants. First, the largest degree of gene expression changes in both leaves and roots occurred at 24 HPT (1,834 and 2,577 up- and downregulated DEGs, respectively, in the leaf; and 2,389 and 2,911 up- and downregulated DEGs, respectively, in the root, Figure 5). The distribution of upregulated and downregulated DEGs identified in the leaves and roots in each cluster is shown in Table 1, and the results of the distribution corresponding to the trend map are shown in Figure 3. The observation that the number of DEGs increase as the treatment time increases indicates a time-dependent traditional reprogramming. In addition, most DEGs at 24 HPT were time-dependent as they were uniquely differential-expressed at this time point (750 and 946 up- and downregulated DEGs, respectively, in the leaf; and 1,432 and 1,447 up- and downregulated DEGs, respectively, in the root). More DEGs in the root compared to the leaf at 24 HPT also indicate that the root responds more strongly to heat stress.

**TABLE 1:** Distribution of DEGs among different clusters.

	Cluster												
	1	2	3	4	5	6	7	8	9	10	11	12	
Genes	1075	1881	2659	3508	4410	1688	2177	3442	1522	3617	2230	1040	Number
Leaf Up-regulated DEGs	89	517	8	230	164	355	418	2	21	1101	179	406	3490
Leaf Down-regulated DEGs	112	11	1006	88	674	20	21	1351	327	18	191	19	3838
Root Up-regulated DEGs	6	386	679	36	5	345	277	1	124	1493	95	344	3791
Root Down-regulated DEGs	241	3	535	14	980	32	8	1213	241	2	192	19	3480

### Heat shock factor enrichment analysis

**FIGURE 6:**
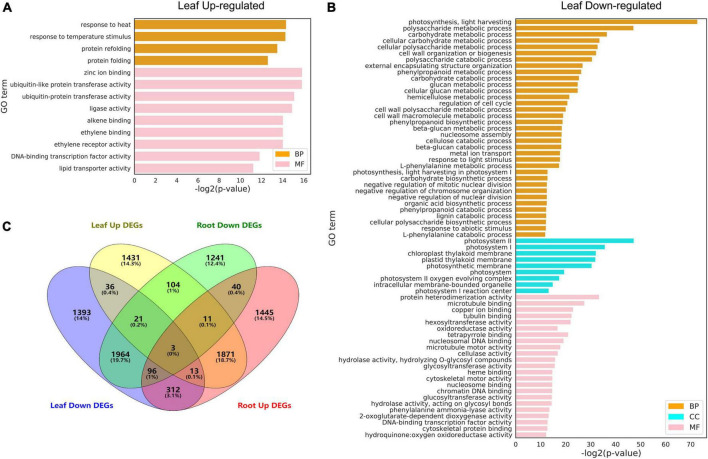
Gene ontology (GO) analysis of differentially expressed genes (DEGs) under heat stress. **(A)** Upregulated DEGs in the leaf. **(B)** Downregulated DEGs in the leaf. **(C)** Venn diagrams showing DEGs in the leaf and root. GO terms with FDR < 0.01 are shown. The full lists of DEGs and GO terms can be found in Supplementary Tables 6, 7.

**FIGURE 7:**
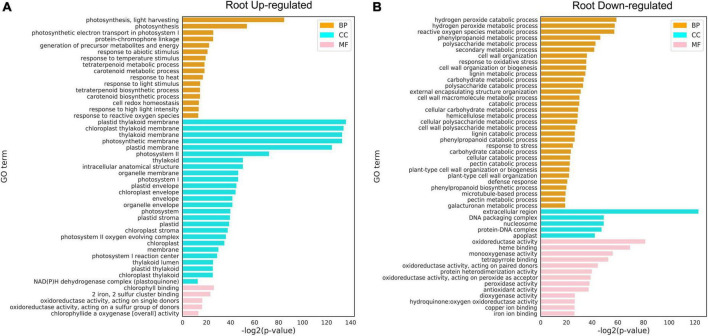
Gene Q20 ontology (GO) analysis of differentially expressed genes (DEGs) under heat stress. **(A)** Upregulated DEGs in the root. **(B)** Downregulated DEGs in the root. GO terms with FDR < 0.01 are shown. CC: cellular component; MF: molecular function; BP: biological process. The full lists of DEGs and GO terms can be found in Supplementary Tables 8, 9.

Gene ontology analysis was performed to functionally annotate the DEGs in the leaf (Figures 6A,B) and root (Figures 6A,B) and the overlapping patterns (Figure 6C). In the leaf, upregulated DEGs were involved in cellular responses to various stimuli including heat and ethylene binding (Figure 6A), while downregulated DEGs were overrepresented in metabolic processes such as polysaccharide metabolic process, cell wall organization or biogenesis, regulation of cell cycle, and photosynthesis I/II. This is in line with the fact that high temperature activates a subset of genes in the leaf to regulate heat stress response and inhibits general metabolism by downregulating metabolism-regulated genes (Saibo et al., 2009). Since there were more DEGs identified in roots than in leaves, there were more GO terms identified in the root. Upregulated DEGs in the root were enriched in cellular components related to photosynthesis (chloroplast, membrane, photosystem I/II, thylakoid lumen, plastid thylakoid, and chloroplast thylakoid; Figure 7A). Downregulated DEGs were enriched in biological processes, such as “hydrogen peroxide catabolic process,” “reactive oxygen species metabolic process,” “phenylpropanoid metabolic process,” “polysaccharide metabolic process,” and “secondary metabolic process” (Figure 7B).

**FIGURE 8:**
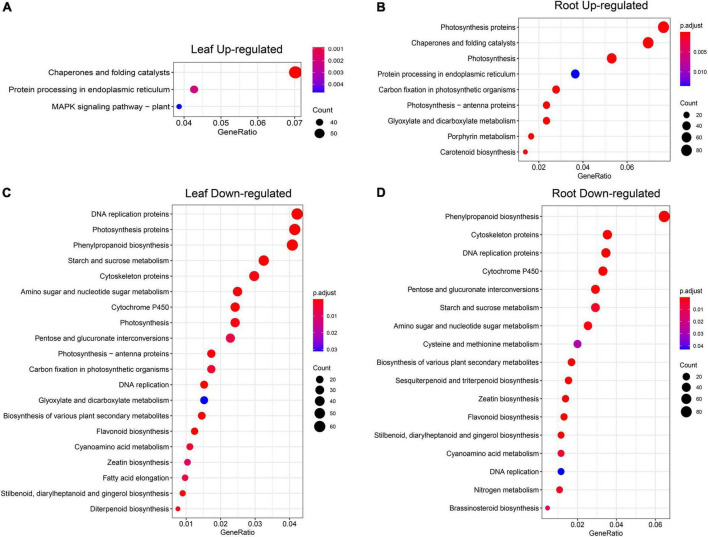
KEGG pathway analysis of DEGs under heat stress. **(A)** Upregulated DEGs in the leaf. **(B)** Upregulated DEGs in the root. **(C)** Downregulated DEGs in the leaf. **(D)** Downregulated DEGs in the root. The full lists of pathway result in Supplementary Table 10.

To further explore on which pathways the up- and downregulated DEGs identified were mainly enriched, we compared the upregulated DEGs (Figures 8A,B) and the upregulated DEGs KEGG pathway analysis was performed on downregulated DEGs (Figures 8C,D), and we found that the number of pathways enriched in downregulated DEGs (Figures 8C,D) was much higher than that in upregulated DEGs (Figures 8A,B). The upregulated DEGs in leaves are enriched in the “MAPK signaling pathway,” which has been shown in previous studies to be closely related to plant response to stress (Jagodzik et al., 2018). The downregulated DEGs in leaves were enriched in some key metabolic pathways, such as “phenylpropanoid biosynthesis,” “starch and sucrose metabolism,” “amino sugar and nucleotide sugar metabolism,” “glyoxylate and dicarboxylate metabolism,” and “flavonoid biosynthesis.” The specific pathways enriched in root DEGs were “flavonoid biosynthesis” and “brassinosteroid biosynthesis” and other important metabolite pathways.

### Phylogeny of the heat shock factor gene family and expression of capsicum annuum heat shock factors

**FIGURE 9:**
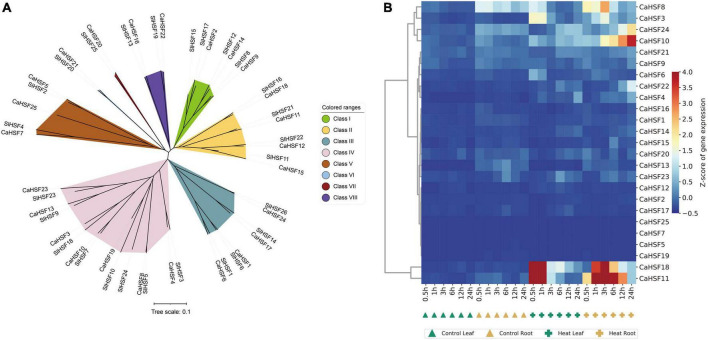
Phylogeny and gene expression of HSFs. **(A)** Phylogenetic analysis of tomato and pepper HSF proteins shows that they can be grouped into eight classes. **(B)** Heatmap showing the temporospatial expression of CaHSFs.

Heat shock factors play a key role in plant heat stress (Scharf et al., 2012) and have also been identified at the genome-wide level in pepper (Guo et al., 2015) and tomato (Yang et al., 2016), but seldom systematically the study of HSFs genes in pepper after heat stress. Our datasets can comprehensively analyze the expression of CaHsfs in pepper of 42°C heat stress at six time points. A phylogenetic tree was constructed to understand the evolutionary relationship among these genes, which were grouped into eight subfamilies (Figure 9A). Interestingly, the expression levels of six CaHSFs (*CaHSF3*, *CaHSF8*, *CaHSF10*, and *CaHSF24* in the leaf; *CaHSF11* and *CaHSF18* in both tissues) were significantly higher in the heat-treated samples compared to control, indicating that heat may activate the expression of these HSFs in pepper (Figure 9B and Supplementary Table 11).

**FIGURE 10:**
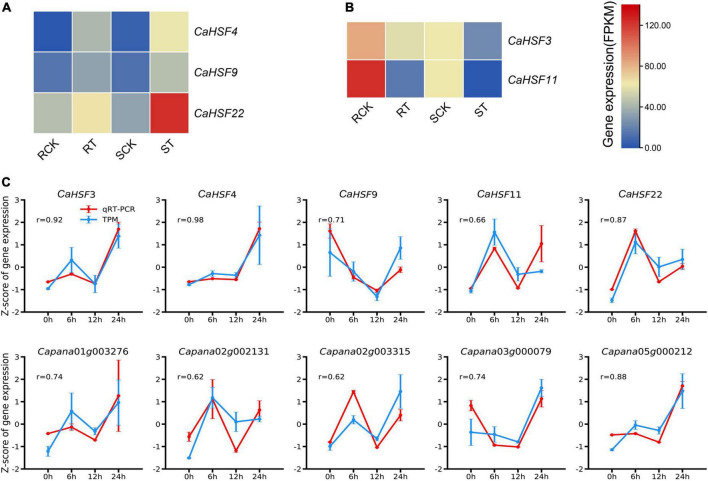
Expression of selected key genes in different pepper varieties. **(A,B)** Expression of *CaHSFs* in heat-tolerant variety 17CL30 and heat-susceptible variety 05S180 after treated at 40°C for 28 h. RCK: control 17CL30; RT: treated 17CL30; SCK: control 05S180; ST: treated 05S180. **(C)** RNA-Seq and qRT-PCR data of the expression profiles of ten selected genes in pepper line 6421 leaves after treated at 42°C for 0, 6, 12, and 24 h. Error bars represent the standard deviation (*n* = 3). *r* = Pearson’s(r) correlation coefficient to describe the correlation between normalized expression values measured by RNA-Seq and qRT-PCR (normalized relative to *CaUBI3*).

To validate these findings, we further analyzed data from a previous study in which the expression of HSFs was assessed in a heat-resistant variety (17CL30; RCK and RT for control and heat treatment, respectively) and a heat-susceptible variety (05S180; SCK and ST for control and heat treatment, respectively) (Wang et al., 2019). In both varieties, three HSFs (*CaHSF4*, *CaHSF9*, and *CaHSF22*) showed higher levels of expression at 28 h of heat treatment at 40°C compared to those in control (Figure 10A), indicating that they could play a role in heat tolerance. By contrast, *CaHSF3* and *CaHSF11* showed opposite expression trends following heat treatment (Figure 10B). To verify the accuracy of the RNA-Seq data and the reproducibility of gene expression patterns, the five CaHSFs (Figures 10A,B) and five randomly selected DEGs from the GO term of “response to heat” (Figure 6A) were analyzed by qRT-PCR, with *CaUBI3* as the housekeeping gene control. The results indicated that the gene expression profiles produced by RNA-Seq and qRT-PCR were in good agreement, with Pearson’s correlation coefficient in the range of 0.62–0.98 among the ten genes. These results confirmed that RNA-Seq in this study produced reliable data on the pepper gene expression profiling.

## Discussion

Understanding the dynamic processes of gene regulation at the genome-wide level when plant is under HS, and identification of key candidate genes that may play important roles in such processes have been the core areas of research. In this study, we performed RNA-seq analysis in both the leaf and root of pepper plants under heat stress at six time points (0.5 to 24 h) and found 12 gene clusters based on the expression profile (Figure 3). In addition, to eliminate the differences in gene expression due to the factors related to circadian clock [i.e., genes may express differentially in day and night (Yu, 2018; Creux and Harmer, 2019)], a set of plants without heat treatment was prepared, from which samples were collected as control at each time point. Furthermore, we selected clusters 2 and 6 from these 12 clusters and performed GO analysis on the genes in these clusters, respectively, and found that they were involved in ethylene binding, molecular transducer activity, ligase activity, signaling receptor activity, response to toxic substances, and oxidoreductase activity, showed obvious enrichment in these GO terms (Figures 4A,B), and a total of 198 transcription factors were identified from these two clusters, of which MYB type transcription factors were the most, with 25, while six HSF transcription factors were also identified. The expression patterns of the transcription factors identified in these two clusters are consistent with the dynamic expression of clusters 2 and 6 in Figure 3, and the response to heat stress is obvious.

Comparative analysis also revealed that the most drastic gene expression changes occurred at 24 HPT following heat stress in both tissues, although the response in root was earlier in time and stronger in magnitude (Figure 5). Thus, our data provide important insights for follow-up mechanistic studies to understand the signal transduction events during stress responses in pepper.

Time-course transcriptome analysis also grouped these genes into 12 clusters, in which the expression of each gene was clearly presented (Supplementary Table 2), and is divided them into different clusters by the difference in the dynamic expression pattern of each gene, which can more clearly show how many different dynamic expression conditions are presented in leaf and root at the whole gene level in pepper under heat stress. Furthermore, through the GO enrichment analysis of cluster 2 and cluster 6, which obviously responded to heat stress, it was found that 1,881 genes in cluster 2 were mainly enriched in terms of molecular function type (Figure 4A) and were mainly enriched in “ethylene binding” and “ethylene receptor activity,” the expression of genes with cluster 2 expression pattern began to increase 3 h after heat stress in pepper, reached a peak at 6 h, and maintained higher levels of gene expression than control tissues, while ethylene is an important plant hormone that regulates multiple developmental and physiological processes throughout the life cycle of plants (Wang et al., 2006), while genes within cluster 2 may be effects on ethylene binding and signaling following heat stress, and transcription factors identified in cluster 2 (Figure 4C) may be involved in these functions. Interestingly, an ortholog of the potential rice thermoreceptor *TT3.1* (Zhang et al., 2022) was found in the cluster 2. This gene was named *CaTT3.1*, and its protein sequence is highly conserved with that of *TT3.1* (Figure 4E). At 3 HPT, *CaTT3.1* was significantly upregulated. It is speculated that *CaTT3.1*, as its counterpart in rice, carries the function of high-temperature sensing in peppers, which may be important for subsequent initiation of heat resistance responses and alleviation of heat damage. Clusters 2 and 6 may contain other genes related to high-temperature response. However, since the gene clusters we have constructed predicted gene functions based on gene expression trends rather than functional studies, the real functions of these genes are yet to be further experimentally confirmed.

Furthermore, GO analysis revealed that DEGs are enriched in heat response and other fundamental biological processes such as photosynthesis, redox regulation, and sugar metabolism. Further KEGG pathway analysis was performed on the upregulated and downregulated DEGs identified in Figure 5 in leaf and root (six time points), and it was found that the upregulated genes were enriched with unique “MAPK signaling pathway,” “chaperones and folding catalysts,” “protein processing in endoplasmic reticulum,” “porphyrin metabolism,” and “carotenoid biosynthesis,” these results indicate that the expression of these genes is upregulated after heat stress treatment, which may make these pathways active in pepper, thereby responding to heat stress excited. Among them, studies have shown that mitogen-activated protein kinase (MAPK) modules play critical roles in the transduction of environmental and developmental signals (Jagodzik et al., 2018). A lot of studies in plants have described the role of endoplasmic reticulum (ER) stress signaling and UPR in regulation of HSFs and HSPs (Hayashi et al., 2012; Mittler et al., 2012; Sandhu et al., 2021). We found that upregulated DEGs in leaf and root (Figures 8A,B) were all enriched in the pathway “Protein processing in endoplasmic reticulum,” which suggests that the expression of genes related to protein processing in the endoplasmic reticulum is significantly upregulated when pepper is subjected to heat stress, which may be involved in the process of pepper’s response to heat stress. Furthermore, leaf genes clustered in the GO term “photosynthesis, light harvesting” were downregulated (Figure 6A). In contrast, root genes clustered in the term were upregulated (Figure 7A). These results indicated that photosynthesis genes in leaves and roots were regulated by HS differently. Understanding the effects of HS on the fate and functionality of chloroplast is particularly important because photosynthesis is usually inhibited by HS before other cellular functions are influenced (Zhang et al., 2010; Ning et al., 2021). Thus, our dataset provided a rich source for follow-up studies on gene regulatory networks under heat in plants.

Interactions between HSFs and HSPs play a key role in regulating adaptation to heat in plants (Driedonks et al., 2015). As the HSR is highly conserved (Richter et al., 2010; Meyer and Baker, 2011; Mittler et al., 2012), HSFs had been identified and analyzed in many plants (Guo et al., 2016). Here, we identified CaHSFs in the Zunla_1 genome (Qin et al., 2014). We found that the expression of multiple CaHSFs (*CaHSF3*, *CaHSF8*, *CaHSF10*, *CaHSF11*, *CaHSF18*, and *CaHSF21*) was inducible by heat in pepper plants. The heat responsiveness of HSF genes was further supported by a previous study, in which the expression of these genes can be either activated (*CaHSF4*, *CaHSF9*, and *CaHSF22*) or suppressed (*CaHSF3* and *CaHSF11*) by heat (Figure 10; Nover et al., 2001). The discrepancy of regulation in *CaHSF3* and *CaHSF11* could be attributed to differences in the genetic background of varieties used in these studies, as an elite breeding line of C. annuum (Line, 6421) was used here and 17CL30 and 05S180 (heat-tolerant and heat-susceptible, respectively) were used in the previous study. Nevertheless, the role of HSFs in heat stress and other abiotic stress had been established in other plants including tomato (Scharf et al., 1998; Wang et al., 2015; Fragkostefanakis et al., 2016). Thus, our finding on the potential role CaHSFs in mediating heat stress in pepper deserves further investigation.

## Conclusion

In summary, we constructed a gene expression network in seedlings of the pepper line 6421 in response to HS and identified genes specifically upregulated in leaves and roots based on K-means analysis of gene expression at the genome-wide level. It is worth emphasizing that the *CaTT3.1* gene, the ortholog of *TT3.1* identified in rice, was captured in Cluster 2, in which all genes showed a significant increase in expression 6 h after heat treatment. In addition to *CaTT3.1*, we also found five CaHSFs whose expression was significantly upregulated after heat treatment. Our study provided important data for better understanding the effect of HS on pepper at a genome-wide level and identified candidate genes that can be potentially used as reference for the subsequent molecular improvement of heat-resistant traits in pepper or other crops.

## Data availability statement

The original contributions presented in this study are publicly available. This data can be found here: https://ngdc.cncb.ac.cn/, CRA006985.

## Author contributions

LX, XL, XZ, and QY: experiment. BT: data curation. FL, XD, and XZ: funding acquisition. BT: visualization. BT: writing—original draft. BT and FL: writing—review and editing. All authors contributed to the article and approved the submitted version.

## Footnotes

1
http://itak.feilab.net/cgi-bin/itak/index.cgi

2
https://www.ncbi.nlm.nih.gov/

3
https://orthomcl.org/orthomcl/

4
https://itol.embl.de/

5
https://meme-suite.org/meme/tools/meme

## Abbreviations

HSFs: heat shock factors
HS: heat stress
h: hours
CaHSFs: capsicum annuum heat shock factors
DEGs: differentially expressed genes
HSPs: heat shock proteins
HSR: heat shock response
GO: gene ontology
BP: biological process
MF: molecular function
KEGG: Kyoto encyclopedia of genes and genomes
FC: fold change
FDR: false discovery rate
FPKM: fragments per kilobase million
HPT: hours post-treatment
TPM: transcripts per million
HCL: hierarchical clustering
PCA: principal component analysis
TFs: transcription factors
ER: endoplasmic reticulum.
